# Intussuscepted Polypoid Meckel’s Diverticulum Presenting With Gastrointestinal Bleeding in a Young Adult

**DOI:** 10.7759/cureus.51744

**Published:** 2024-01-06

**Authors:** Christos Konstantakis, Petros Mantzios, Christos Sotiropoulos, Stathis Anesidis, Konstantinos C Thomopoulos

**Affiliations:** 1 Gastroenterology Department, University General Hospital of Patras, Patras, GRC; 2 General Surgery Department, University General Hospital of Patras, Patras, GRC

**Keywords:** gastrointestinal bleeding, small bowel capsule endoscopy, intussusception, inversion, meckel’s diverticulum

## Abstract

A 22-year-old female patient with a recent hospitalization for gastrointestinal bleeding presented with recurrent hematochezia and a positive shock index. Previous investigations, including endoscopy and wireless small bowel capsule, were non-diagnostic. CT angiography revealed extravasation in the ileum. Initial tests like technetium-99m scintigraphy and ileocolonoscopy were negative. Repeat wireless small bowel capsule identified a partially ulcerated polypoid mass in the distal ileum. At surgical exploration, an intussuscepted Meckel’s diverticulum was identified and resected. A histopathologic examination confirmed the diagnosis. Meckel's diverticulum is a rare cause of gastrointestinal bleeding in adults. Preoperative diagnosis can be challenging. Reports of a polypoid morphology are very scarce in indexed literature and mostly derive from investigation with device-assisted enteroscopy. We report this extremely rare finding at capsule endoscopy to raise clinician awareness and to discuss diagnostic difficulties associated with similar cases, such as the negative scintigraphy result and the optimal timing of repeat capsule endoscopy.

## Introduction

Meckel’s diverticulum (MD) is the most prevalent congenital malformation of the gastrointestinal (GI) tract [1]. Ιt is an embryologic remnant of the midgut usually containing ectopic gastric tissue (16-50%) and more rarely ectopic pancreatic tissue (5%) [1,2]. MD inversion is a rarely encountered entity and most information about it derives from case reports. MD-related symptoms in children usually present within the first decade of life and include GI bleeding, anemia and, to a lesser extent, abdominal pain [1,2]. Surgical resection is the treatment of choice for symptomatic MD, while management of incidentally diagnosed MD is still controversial [1,2]. Resection of asymptomatic MD is usually indicated in the presence of certain risk factors for long-term complications, such as male sex, young age (<45 years), diverticulum length >2cm and the presence of ectopic mucosa on rapid biopsy [1].

Lifelong risk of complications ranges between 4-6% [2]. Since complications arising from MD are much more prevalent in children, they are erroneously overlooked in the differential diagnosis of GI bleeding and abdominal pain in the adult population. Wireless capsule endoscopy (WCE) is recommended by endoscopic societies’ guidelines as the modality of choice for evaluating the small intestine in cases of obscure GI bleeding [3].

Herein, we present the case of a young adult with recurrent hematochezia due to an intussuscepted, inverted MD, diagnosed via repeat WCE after an extensive negative initial work-up.

## Case presentation

A 22-year-old female patient with recent hospitalization in another regional institution for lower GI bleeding presented to our hospital with recurrent hematochezia 6 weeks after discharge. Previous work-up including both upper and lower GI endoscopy and investigation of the small intestine with wireless capsule endoscopy (WCE) failed to reveal the source of bleeding. The degree of cleanliness of the small bowel was classified as fair overall with poor preparation of the distal ileum. Abdominal CT scan (with oral and IV contrast) and a magnetic resonance enterography (MRE) were also non-diagnostic. Conservative treatment was chosen. Following symptom resolution, the patient was discharged. Patient history revealed a chronic iron deficiency anemia treated with oral iron supplements. The patient was a non-smoker with no pharmaceutical allergies.

The patient presented at our emergency department with pallor, positive digital rectal examination for blood and hemodynamic instability with a positive shock index [4]. She was afebrile (T=36.4 ^o^C), with blood pressure measured at 98/60 mmHg and heart rate (HR)= 110 bpm. At physical examination, bowel sounds were mildly increased and slight tenderness during palpation in the lower abdomen was noted, without guarding or rebound. Complete blood count (CBC) revealed severe normocytic anemia (hemoglobin-Hgb= 6.9 g/dL, hematocrit-Hct= 20.8%, mean corpuscular volume-MCV=89 fl) mild thrombocytopenia (platelets-PLT=114.000) and mild lactate dehydrogenase increase (LDH=270), while white blood cells, coagulation parameters and biochemical markers, including liver function tests and inflammatory markers (erythrocyte sedimentation rate-ESR, C-reactive protein-CRP), were within the normal range (Table 1). 

**Table 1:** Patient blood tests on admission. Pathologic results are presented in bold.

Lab parameter	Value	Reference ranges	Units
Hemoglobin (Hgb)	6.9	12-15 (for females)	g/dL
Hematocrit (Hct)	20.8	38-48 ( for females)	%
Mean corpuscular volume (MCV)	89	79-98	fL
Total Leukocytes-WBC	8.90	4-11	K/μL
Neutrophils (absolute count)	6.6	1.8-7	K/μL
Lymphocytes (absolute count)	1.85	1.2-3.8	K/μL
Monocytes (absolute count)	0.39	0.2-0.8	K/μL
Neutrophils (%)	74.6	50-70	%
Lymphocytes (%)	20.6	20-40	%
Monocytes (%)	4.3	2-8	%
Platelets (PLT)	114	140-450	K/μL
aspartate aminotransferase (AST)	37	0-40	U/L
alanine aminotransferase (ALT)	22	<45	U/L
Αlkaline Phosphatase (ALP)	28	24-120	U/L
Gamma-glutamyl transferase (γ-GT)	7	10-49	U/L
Lactate dehydrogenase (LDH)	270	120-230	U/L
Creatine phosphokinase (CPK)	112	<190	U/L
Total Bilirubin	1.1	< 1.2	mg/dL
Direct Bilirubin	0.2	<0.3 mg/dl	mg/dl
Albumin	3.6	3.4-4.8	g/dL
Creatinine	0.7	0.7-1.4	mg/dL
Urea	39	10-50	mg/dL
Glucose	106	75-115	mg/dL
Sodium (Na^+^)	138	136-145	mmol/L
Potassium (K+)	4.5	3.5-5.2	mmol/L
C-reactive protein (CRP)	0.04	<0.7	mg/dL
Erythrocyte Sedimentation Rate (ESR)	10	<30	mm/Hr
International normalized ratio (INR)	1.09	0.8-1.1	-
Prothrombin time (PT)	12.0	9.1-12.1	sec
Activated partial thromboplastin time (aPTT)	28	24-36	sec

**Figure 1:**
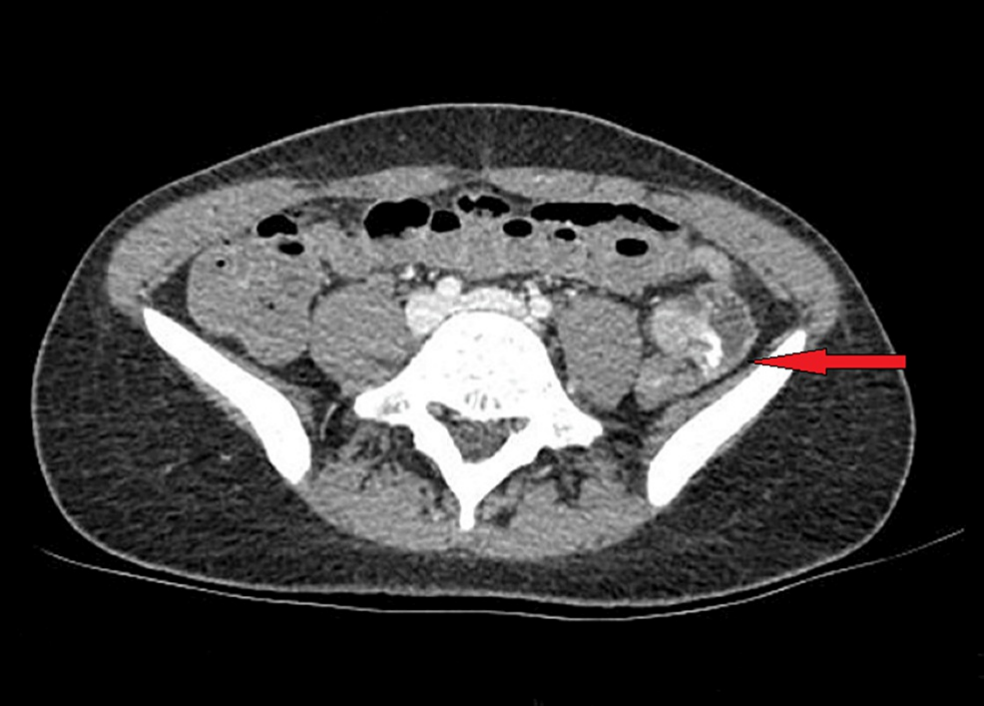
Abdominal CT angiography depicting contrast extravasation in the distant ileum (indicated by red arrow)

The patient underwent CT angiography on admission, which suggested contrast extravasation in the distal ileum (Figure 1). No pathologically enlarged lymph nodes or free fluid in the peritoneal and pelvic cavity were detected. Interventional radiologists were consulted for emergency embolization. However, at the time of angiography, active bleeding was not confirmed. Hence, we opted against performing a non-super-selective embolization to a patient of this age, in view of possible complications, namely bowel ischemia. Instead, our patient was supported with transfusions of two packs of fresh frozen plasma (FFP) and three units of packed red blood cells (RBC).

**Figure 2:**
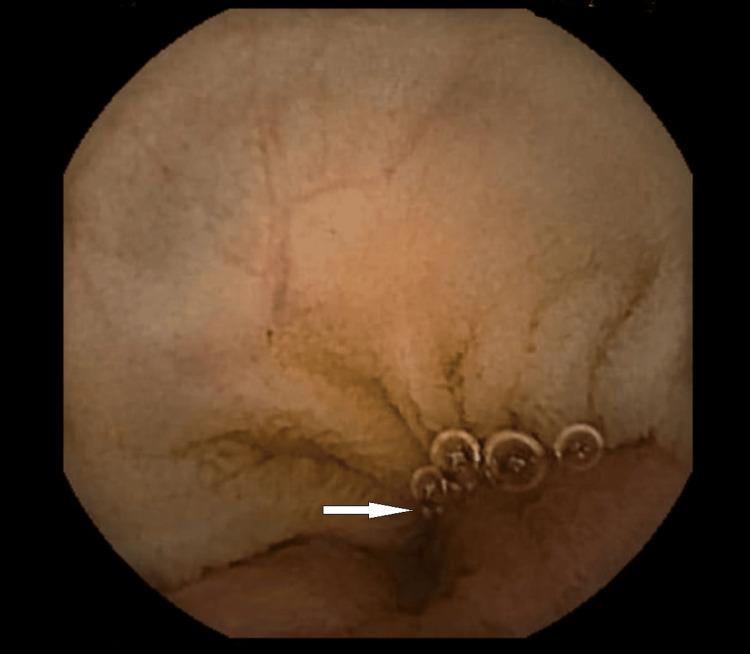
Endoscopic capsule image of an inverted polypoid Meckel's diverticulum (indicated by white arrow)

**Figure 3:**
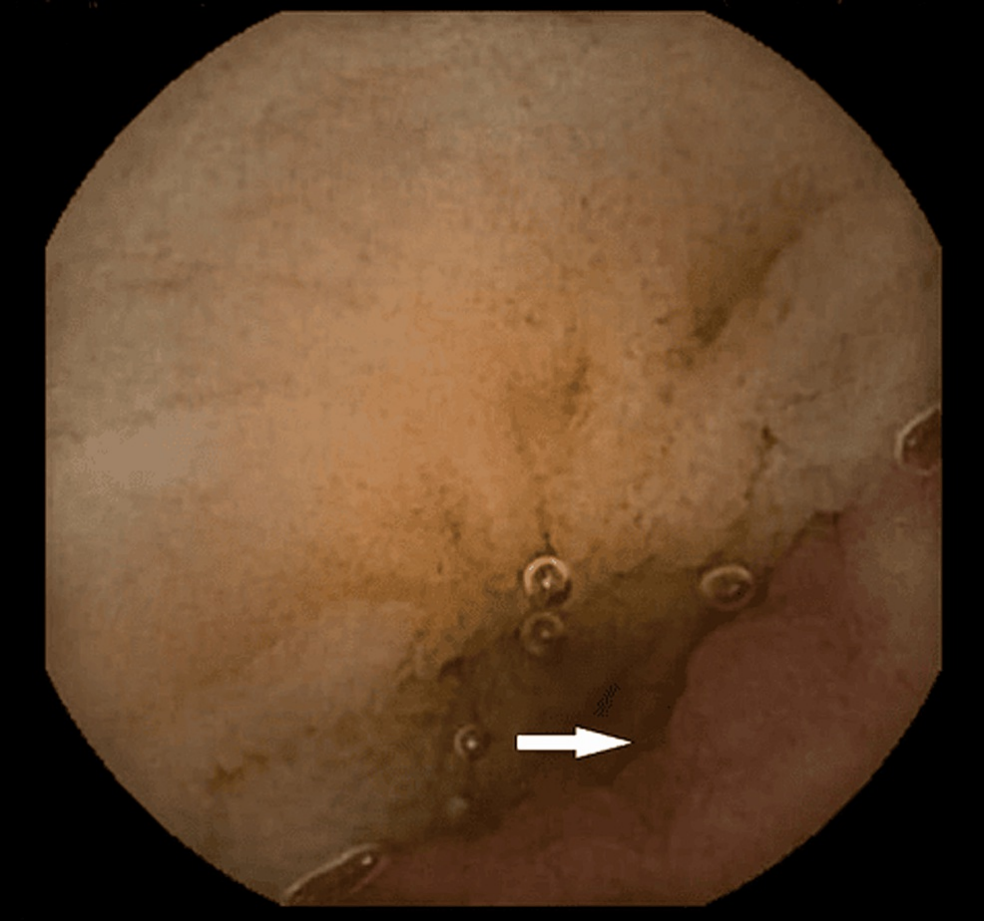
Endoscopic capsule image of an inverted polypoid Meckel's diverticulum (indicated by white arrow)

Following clinical stabilization, improvement of anemia and remission of active bleeding, she underwent a Technetium-99m scintigraphy on the third day after admission and complete ileocolonoscopy the following day. Neither exam reported pathological findings. Blood clots were observed in the colon lumen, without identifying a specific bleeding site. Subsequently, our patient was scheduled for a repeat WCE after adequate bowel preparation with 2 litres of polyethylene glycol solution (PEG). Approximately 12 min. from the terminal ileum, an abnormal, blue-violet lobular mass without signs of active bleeding was detected, projecting into the intestinal lumen (Figures 2, 3).

**Figure 4:**
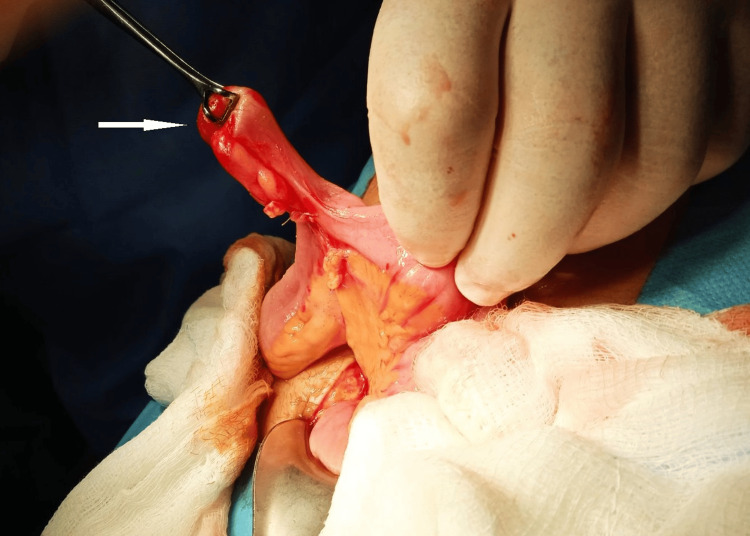
Surgical resection of the intussuscepted Meckel's diverticulum (indicated by white arrow)

The patient was transferred to the surgical clinic and underwent an exploratory laparotomy. At 60 cm from the terminal ileum, an intussuscepted, inverted Meckel’s diverticulum (MD) was identified (Figure 4). Segmental resection and primary enteric anastomosis followed afterwards. The diagnosis was supported both by the rapid biopsy and the final surgical preparation (confirming the presence of heterotopic fundus mucosa with areas of superficial erosion and ulceration). The patient recovered uneventfully post-surgery and was discharged three days later. On follow-up, she remains in excellent clinical condition, without symptom recurrence.

## Discussion

MD is a congenital abnormality of the GI tract and embryologically stems from deficient involution of the omphalomesenteric duct, a process normally completed by the 7th week of gestation [1,2]. It is a true diverticulum (a blind pouch containing all four GI wall layers) and is usually located on the antimesenteric border of the ileum, within 60-100 cm from the ileocecal valve [2]. It is more commonly encountered in males (male: female ratio = 3:1) and the overall incidence is estimated between 0.4-3% [1,2,5]. It is often incidentally recognized during autopsy, since in 98% of cases it causes no symptoms throughout a person’s lifetime [2].

Inverted MD is an unusual anatomic variation; little more than 100 cases have been reported in world literature, most of them in recent years [6,7]. The pathophysiology of its formation is not fully understood, however chronic abnormal peristalsis caused by the ectopic tissue located at the base of the MD is the most likely mechanism [1,8]. An inverted MD may act as a lead point for small bowel intussusception [9]. This represents the most common manifestation in adults, while bleeding episodes are far less frequent, in contrast to pediatric patients.

Ulcer formation is usually attributable to gastric acid secretion by the ectopic parietal cells, damaging the much more susceptible, exposed surrounding intestinal tissue [1]. When gastric tissue is not present, erosion is explained by repeated episodes of intermittent, incomplete intussusception, causing mechanical injury and tissue ischemia [1,2,8]. Our patient met both those conditions and had been diagnosed with iron deficiency anemia, before presenting with recurrent episodes of red dark stool.

In our case, MD was detected after repeat WCE, which is a non-invasive modality that allows detailed visualization of the intestinal mucosa when high-quality images are obtained [3,10]. We opted for this approach since it is a readily available technique in our referral center, with good patient tolerance. Adequate bowel cleansing is important. WCE is the following step in investigating obscure GI bleeding after negative upper and lower endoscopy work-ups, a practice included in the latest European Society of Gastrointestinal Endoscopy (ESGE) guidelines [3]. Diagnostic yield in cases of GI bleeding increases when WCE is undertaken within 3-14 days of the last bleeding episode [3,10]. Few cases of inverted MD have been diagnosed using this technique, therefore its application regarding this condition is not well studied [11,12]. However, a study by Krstic et al reported a positive predictive value of 84.6 %, suggesting its promising potential [13]. In WCE, the most common finding recognized is the “double lumen sign”, reported to be present in up to 69% of MD cases, followed by the “diaphragm sign” and detection of peripheral ulcers [5,14]. WCE has been approved by the United States Food and Drug Administration (FDA) for use in children older than two years and inflammatory bowel disease (IBD) suspicion is the most common indication [3]. However, some children exhibit an inability or unwillingness to properly swallow the capsule. Moreover, WCE cannot provide endoscopic treatment when pathology is identified and is contraindicated when intestinal obstruction is suspected [3]. The most serious complication is retention of the capsule in the diverticular lumen, however, it is quite uncommon (<2% of cases) and this possibility should not solely guide clinical decision-making [14].

Various imaging modalities are available in clinical practice to diagnose MD complications, each with distinct advantages and limitations. Plain abdominal radiography can be helpful in diagnosing non-specific signs of intestinal obstruction [2]. Ultrasound is usually the primary imaging modality in children, but its value in adults is limited [2]. Screening withTc-99m scintigraphy (also known as a Meckel’s scan) is considered the test of choice for confirming MD diagnosis in cases of lower GI bleeding. However, its result may be negative despite the presence of ectopic gastric mucosa, as in our patient. This could be attributed to mucosal ischemia due to intussusception and thus reduced radioactive tracer uptake by the mucin-secreting cells [2,8]. Furthermore, Meckel's scan is less reliable in adults compared to the pediatric population (accuracy of 55-60% compared to 90%, respectively) [2,8]. Therefore, it lacks negative predictive value and a negative result cannot safely exclude an underlying MD diagnosis in adult patients.

CT enterography (CTE) has demonstrated superior sensitivity compared to conventional CT with regards to detecting mural abnormalities and helping an experienced radiologist differentiate MD from normal bowel loops. Nevertheless, it exposes the patient to high doses of radiation [2]. MRE combines the lack of ionizing radiation with enhanced soft tissue contrast and the capability of three-dimensional imaging reconstruction, rendering it a sensitive and attractive imaging technique which is increasingly utilized [2]. While often helpful in identifying MD, even in cases of inversion, MRE did not contribute to the diagnosis in our patient. Double balloon enteroscopy (DBE) and concurrent retrieval of biopsy specimens has demonstrated promising efficacy in recognizing and verifying MD diagnosis in recent case series [15,16]. It also enables hemostatic intervention in cases of severe bleeding and is more cost-effective in comparison to WCE [15]. However, examination of the whole small intestine through DBE is a technically demanding procedure which is not widely available in all hospitals, including our institution. Instead, WCE provided the diagnosis and the patient was scheduled for an exploratory laparotomy, which allowed for concurrent treatment.

## Conclusions

We presented the case of a young adult with recurrent hematochezia caused by an intussuscepted, inverted MD. Even in the presence of ectopic gastric mucosa, an intussuscepted MD may not be detectable with Tc-99m scintigraphy due to impaired perfusion and radiotracer uptake. Therefore, the threshold for repeat capsule endoscopy, especially when there is a strong clinical indication, should be low. This is in line with recent endoscopic guidelines. This inverted MD did not present with the typical endoscopic finding of a “double-lumen” but as a protruding polypoid mass. This is a rarely described image in capsule endoscopy and we hope it contributes to raising awareness among clinicians towards this rare cause of lower GI bleeding in adults.
